# Now You See It. Now You Don’t. Who Can Tell Where the Little Joker Is?

**Published:** 1910-05

**Authors:** 


### "NOW YOU SEE IT. NOW YOU DON'T. WHO CAN TELL WHERE THE LITTLE JOKER IS?"

AUSTIN, TEXAS, April 25, 1910.

*Mr. Frederick Gree, Assistant to the General Secretary, A. M. A., Chicago.*

DEAR SIR: I have the honor to acknowledge receipt of your letter in reply to my inquiry, in which you state that "the cost of membership in the American Medical Association is five dollars per year." I see an official announcement in a late copy of the *Journal of the A. M. A.* as follows: "The annual assessment for membership shall be one dollar, and the subscription price of the *Journal* shall be four dollars for members." Having recently received an invitation from Secretary-Editor Simmons to become a member and a subscriber, accompanied by the printed statement that a subscription *"carries with it membership in the Association,"* I am somewhat confused by these conflicting statements and I must respectfully ask for information, if you please. Which is correct? Shall I send five dollars for subscription and receive membership as a *premium*, or shall I send five dollars for membership, as per your letter, and receive the *Journal* as a premium, free; or shall I send one dollar for membership, omitting the *Journal;* or is taking the *Journal* obligatory on all who join? I will greatly appreciate your early attention.

Yours respectfully,

F. E. Daniel.

Note.—I wish to publish this and your reply for the information of such of my readers as may desire to join the A. M. A.

---

Look on this picture and then on that which follows. The bound volume of Transactions for 1904, 650 pages, containing all the papers and discussions, together with all the essentials to keep record of the society, cost the Association just 77 cents per copy. It was delivered free to members, post or express paid. The members care only for the papers and discussions to keep in their library. The *State Journal* publishes them all, maybe, in the *course of the year,* the bulk of each issue being devoted to lists of members of county societies, news of transient interest, etc., not worth preserving; yet, if one cares enough for the files to keep them a year, he can get them bound for $3, or if he returns them to the Secretary *in good order* he can exchange them for a bound volume for $1.50. This, in addition to the $2 he pays annually. See April number of *State Journal,* page 433. At Dallas there will be a resolution offered to make the annual dues $1 and the subscription $1, optional; and another resolution to publish all the papers and discussions *at once* in a bound volume to be furnished free to every member, while the *Journal* can be devoted, as it largely is now, to the news and lists of members and buncombe. And every member should have a vote on this and all other resolutions affecting their rights and interests.

